# Use of Complementary and Alternative Medicine and Adherence to Medication Therapy Among Stroke Patients: A Meta-analysis and Systematic Review

**DOI:** 10.3389/fphar.2022.870641

**Published:** 2022-06-03

**Authors:** Sareneya Dashni Rajahthurai, Muhammad Junaid Farrukh, Mohd Makmor-Bakry, Hui Jan Tan, Omotayo Fatokun, Shamin Mohd Saffian, Diana Laila Ramatillah

**Affiliations:** ^1^ Faculty of Pharmaceutical Sciences, UCSI University, Kuala Lumpur, Malaysia; ^2^ Faculty of Pharmacy, Universiti Kebangsaan Malaysia, Kuala Lumpur, Malaysia; ^3^ Faculty of Medicine, Universiti Kebangsaan Malaysia, Kuala Lumpur, Malaysia; ^4^ Pharmacy Faculty, Universitas 17 Agustus 1945, Jakarta, Indonesia

**Keywords:** adherence, compliance, persistance, complementary and alternate medicine, stroke, diagnosis, CAM, stroke medication

## Abstract

**Purpose:** To identify the use patterns of complementary and alternative medicine (CAM) and its impact on medication adherence among patients with stroke.

**Method:** A systematic search through Science Direct, Google Scholar, and PubMed was performed to identify potential studies up to June 2021.The primary outcome was CAM use, and the secondary outcome was medication adherence among patients with stroke. Articles included in the review met the following criteria: 1) patients with stroke ≥18 years old on prescribed medications, and 2) medication adherence reported status. Meta-analyses were conducted to estimate the pooled prevalence of complementary and alternative medicine and adherence in stroke patients using a random-effects model.

**Results:** A total of 1,330 studies were screened, of which 22 were included in the final analysis. The type of studies included were cross-sectional surveys, cohort studies, retrospective studies and prospective survey. The pooled prevalence of CAM usage was at 38% (29–48% CI) and medication non-adherence among stroke patients was at 29% (20–48% CI). The most common reason for inadequate stroke therapy and higher dependence on CAM was the patients’ lack of knowledge and the regimen complexity of the medication. Other factors for medication non-adherence were forgetfulness, side effects, cost, and lack of doctor-patient communication.

**Conclusion:** A low prevalence of CAM usage and non-adherence to medications was observed among patients with stroke. Studies investigating the association between CAM usage and medication adherence among patients with stroke are scarce and future researches are needed to explore the influence of CAM use on stroke medication adherence.

## Introduction

Stroke is a non-communicable disease and a focal neurological disturbance of vascular origin that lasts more than 24 h(Khaku and Tadi, 2020). According to the World Stroke Organization, 13.7 million new stroke cases are diagnosed each year worldwide (Lindsay et al., 2019) and approximately 80 million people in the world have had a stroke. Overall, Oceania, Southeast Asia, North Africa, the Middle East, and East Asia had the highest age-standardized stroke prevalence rates in 2019. The countries with the highest prevalence rates of ischemic stroke were found in North Africa and the Middle East, southern Sub-Saharan Africa, high-income North America, and Southeast Asia (Virani et al., 2021). Stroke has been intended to become the second largest cause of mortality by 2040 (MOH, 2019). On average, 92 admissions and 32 deaths occur each day across Malaysia’s healthcare facilities (MOH, 2019). The prevalence of stroke in Malaysia was estimated to be 0.3% in 2006 during the third National Health and Morbidity Survey (NHMS). Stroke is a chronic condition characterised by acute events (O’Neill et al., 2008). Thus, long-term therapy is indicated for stroke patients, especially antiplatelet, antihypertensive, and lipid-lowering agents for ischemic stroke and anticoagulants for embolic stroke (MOH, 2012; Linden, 2020). Therefore, adherence is an important factor in determining the clinical outcomes of stroke patients.

Complementary alternative medicine (CAM) is comprised of diverse structures, methods, and products that are not commonly considered part of traditional medicine (Barnes et al., 2008). In Asian and African countries, the type of CAM used to treat stroke may vary due to geographical and cultural differences (Jeong et al., 2016). In the last decade, there has been a rapid rise in interest in CAM among stroke patients. CAMs are commonly categorized into five groups, according to the National Center for Complementary and Alternative Medicine (NCCAM) (2005): biological, spiritual/mind-body, alternative, physical (body-based), and energy therapies (Wieland et al., 2011). CAM for stroke includes acupuncture, herbal remedies, Ayurveda, Chinese medicine, homeopathy, mind-body techniques, and physio (Shin et al., 2008). Studies have suggested that CAM is used worldwide (Aydin et al., 2008; Fox et al., 2010; Shaikh et al., 2009). Herbs, vitamins, herbal additives, enzymes, and minerals are biochemical processes, also known as natural goods, which are biological methods, while wellbeing activities include acupuncture, aromatherapy, cupping, meditation, praying (use of holy water, amulets, or talismans), and Zumba. Traditional medicines that differ from region to region are used in alternative medicine, such as traditional Chinese, Malay, Indian (Ayurvedic/Siddha/Unani) medicines, and homeopathic remedies (Rhodes et al., 2008; Wieland et al., 2011). Due to variations in cultural values and healthcare environments, the CAM use can vary. A survey indicated that 71.2% of respondents use CAM, wherein the use of herbal products was the most frequent. Recommendations from relatives and friends are the key justifications for using CAM (Jasamai et al., 2017).

Medication adherence is defined as the degree to which patients follow their doctor’s instructions and their persistence in fulfilling the span of time from start to discontinuation of therapy. (Shams and Barakat, 2010), (Vrijens et al., 2012) Meanwhile, adherence can also be defined as patients simply not taking their medications. Medication adherence measures are generally categorized as arbitrary and quantitative measurements by the WHO (Sabaté and Sabaté, 2003). Subjective assessments include those that require the appraisal of their medication-taking actions by the patient (Velligan et al., 2007). The most popular instruments used to test opioid adherence include self-reported questionnaires, for example, MMAS, as well as healthcare provider interviews. Quantitative tests include pill counts and secondary review of records, such as MPR, computer tracking, and biochemical measures (Vermeire et al., 2001).

The four primary factors related to medication non-adherence are patients (e.g., socioeconomic characteristics, and perceptions and beliefs), illness-related factors (e.g., severity of illness and frequency of symptoms), medication (e.g., number of daily doses, efficacy, and side effects), and physician-related ones (e.g., patient-physician relationship), (Greenhouse et al., 2000; Scott and Pope, 2002; Kleindienst and Greil, 2004). Medication non-adherence is a severe concern to patients with stroke, and the assumption that stroke has a moral or psychological origin can contribute to insufficient stroke treatment and stronger Dependency on CAM may lead to non-adherence to medication, however, there is a lack of information regarding the influence of CAM on the adherence of patients with stroke. The objectives of the present study were to evaluate patterns and types of CAM use, prevalence of medication adherence and to determine the effect of CAM on medication adherence in patients with stroke.

## Methods

### Data Sources and Searches

Studies were identified through a comprehensive literature search of Embase and Cochrane Library, Science Direct, Google Scholar, and PubMed from the inception of these databases until June 2021. The keywords used for searching for relevant articles were “adherence,” “compliance,” “medication,” “stroke,” “non-adherence,” “CAM,” and “complementary and alternative medicine.” Boolean operators such as “AND” and “OR” were used to increase the sensitivity and specificity of the search. The articles identified were assessed based on the inclusion and exclusion criteria presented in Table 1. This review was restricted to studies published in English only.

**TABLE 1:** Criteria for inclusion and exclusion of studies in the review.

Population	Stroke Patients Aged 18 and Above
Phenomenon of interest	The phenomena of interest included
	• The global of pattern of CAM usage
	• Types and reasons of CAM usage
	• Adherence to stroke medications
Primary outcome measure	The outcome measures of interest included, but were not restricted to the following
	• Prevalence and types of CAM usage
	• Prevalence of adherence and method of assessment
	• Factors associated to medication non-adherence
	• Impact of CAM on adherence and stroke therapy
Types of studies	Quantitative cross-sectional surveys. Studies were included if they reported one or more of the outcomes detailed above

CAM, complementary and alternative medicine.

### Study Selection

The titles and abstracts were screened by SDR for inclusion, and another author, MJF, was consulted. The full texts of relevant articles were downloaded and assessed for inclusion against the inclusion and exclusion criteria by MJF and checked by HJT. Disagreements were resolved by consensus. In instances where consensus was not reached between the two review authors, the third review author, MMB, was consulted.

### Data Extraction

Data about study and participant characteristics and prevalence of CAM use, including the types of CAM used by stroke patients, were extracted. Furthermore, data on medication adherence rate, methods of adherence assessment, and factors that lead to non-adherence were also extracted from the full-text report by the author. The extracted data were grouped into geographical regions and income levels. Countries were categorized into different income level categories as defined by the World Bank.

### Quality Assessment

Three review authors (MJF, MMB, and THJ) independently assessed the quality of included studies using the Critical Appraisal Skills Programme (CASP) checklist. The primary results of the current study were the rate of adherence of patients to stroke drugs, the use of CAM, and the impact of CAM on adherence to stroke drugs. By using the evaluation tool for quantitative research studies, the Critical Appraisal Skills Programme (CASP) tool, the quality of the studies was evaluated for potential bias (Long et al., 2002).

### Statistical Analysis

The meta-analysis was performed using STATA version 15^®^. Clinical homogeneity was assessed by review authors in terms of the study population and methods used to assess medication adherence. I^2^ with a 95% confidence interval test was used to evaluate the statistical heterogeneity of the studies. (Higgins and Thompson, 2002) An analysis of proportions was performed using a random effect model to account for statistical heterogeneity. This is because it is assumed that there is significant heterogeneity among the studies (Barendregt et al., 2013). Furthermore, subgroup analyses based on the level of income of the country and country region were performed to address heterogeneity.

## Results

**FIGURE 1:**
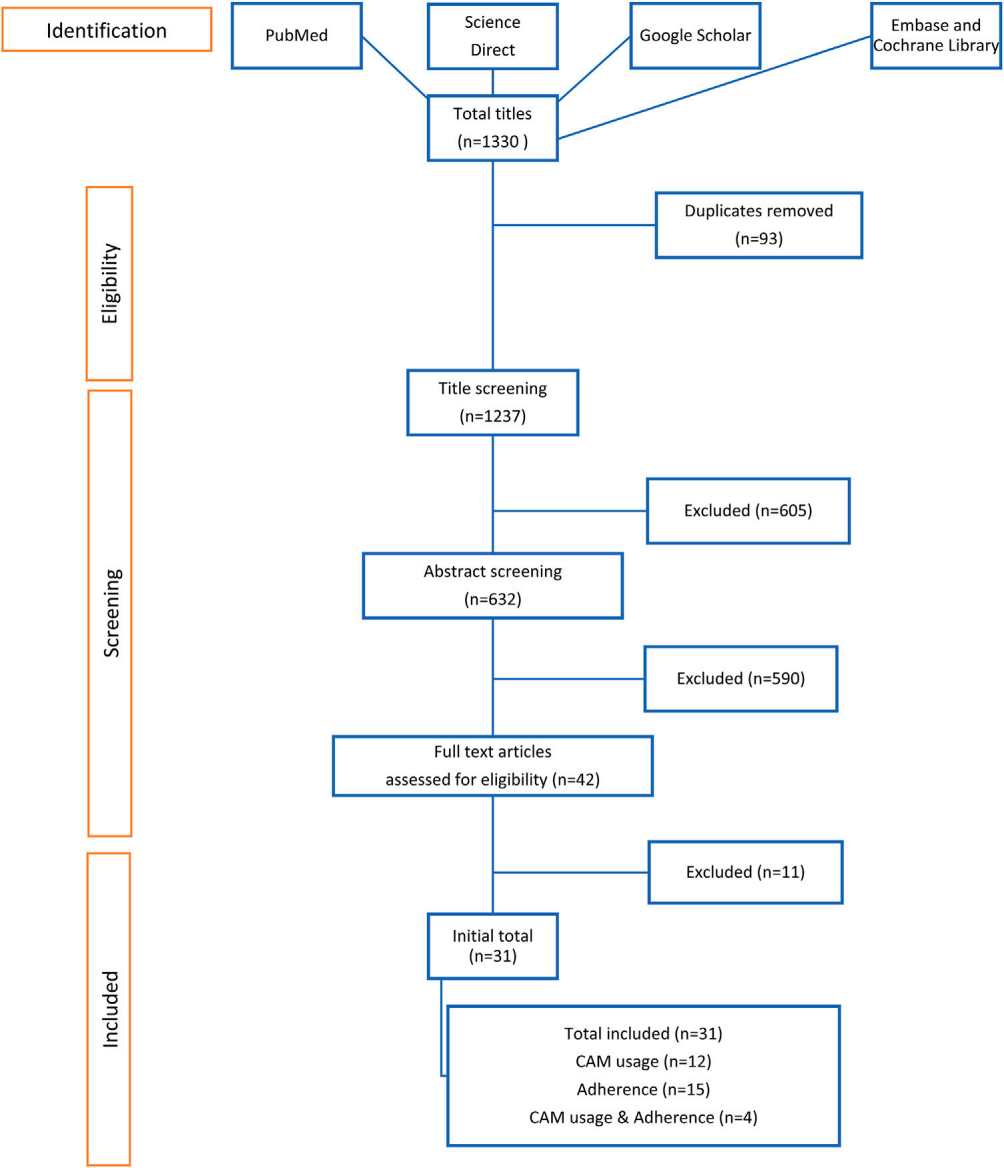
Flow diagram of searches and inclusion assessment of studies.

A total of 1,330 titles were recovered, and 93 duplicates were removed. The remaining papers were screened for inclusion and exclusion criteria. A total of 22 studies were included in the final analysis. The flow of research is shown in Figure 1.

Of the 31 studies, 12 focused on CAM usage among patients with stroke, (Lee et al., 2004; Shin et al., 2008; Shaikh et al., 2009; Chang et al., 2011; Liao et al., 2012; Pandian et al., 2012; Ali et al., 2015; Kadir et al., 2015; Chang et al., 2016; Teo et al., 2016; Weng et al., 2016; Yeh et al., 2017), 15 reported medication adherence, (De Schryver et al., 2005; Osterberg and Blaschke, 2005; Arif et al., 2007; Khan et al., 2010; Bushnell et al., 2011; Chambers et al., 2011; Kronish et al., 2013; Sjölander et al., 2013; Jiang et al., 2017; Kim et al., 2017; Wei et al., 2017; Cheiloudaki and Alexopoulos, 2019; Rohde et al., 2019; Han et al., 2020; Saade et al., 2021), and only 4 investigated CAM usage and patients’ adherence to stroke medications (Johnson et al., 2012; Karuniawati et al., 2017; Alhawsawi et al., 2020; Kim et al., 2020). The study design and country settings are summarized in Table 2, Table 3, Table 4 to Table 5.

**TABLE 2:** Prevalence and types of CAM usage.

No	Authors	Year	Type of Study	Country	Sample Size (n)	CAM Usage (%)	Types of CAM Used n (%)	Mind and Body Practice	Natural Products	Alternative/Traditional Medicine
1	Shin et al. (Shin et al., 2008)	2007	Survey among stroke patients	Korea	304	54	36% Herbal products	√	√	√
							24% Vitamins			
							11% Manual therapies			
							8% Charcoal and oxygen generator			
							7% TCM			
							6% Spiritual			
							1% Bioelectromagnetic therapies			
2	Lee et al. (Lee et al., 2004)	2004	Cross-sectional study	Singapore	539	22.7	37.8% TCM	√	—	√
							29.7% Dietary therapy			
							27.5% Acupuncture			
3	Liao et al. (Liao et al., 2012)	2012	Cross-sectional study	Taiwan	15,330	32	TCM	—	—	√
4	Yeh et al. (Yeh et al., 2017)	2017	Survey among stroke patients	Taiwan	212	62.3	TCM	—	—	√
5	Chang et al. (Chang et al., 2011)	2011	Survey among stroke patients	Korea	2,167	18.2	Acupuncture	√	—	
6	Chang et al.(Chang et al., 2016)	2016	Survey among stroke patients	Taiwan	23,816	12	TCM	—	—	√
7	Weng et al. (Weng et al., 2016)	2016	Cohort study	Taiwan	285,001	17	Acupuncture	√	—	—
8	Teo et al. (Teo et al., 2016)	2014	Cross-sectional study	Singapore	768	43.4	29.4% TCM	√	—	√
							22.3% Acupuncture and Chiropractic			
							11.3% Yoga and Tai Chi			
							1.2% Magnetic therapy			
9	Pandian et al. (Pandian et al., 2012)	2010	Prospective study	India	314	36.3	59.3% Ayurvedic massage	√	√	√
							19.5% NaCl intravenous fluid with vitamin injections			
							15% Herbal medicines			
							13.3% Homeopathic drugs			
							8.8% Multivitamin and mineral supplements			
10	Kadir et al. (Kadir et al., 2015)	2010	Prospective cohort study	Malaysia	93	66.7	36.6% Massage	√	√	√
							97.8% Herbal			
							92.5% Vitamins			
							97.8% Traditional healers			
11	Shah et al. (Shah et al., 2008)	2008	Cross-sectional study	United States	806	46	20.4% Herbal Medicine	√	√	√
							19.4% Chiropractic			
							4.5% Yoga and Tai Chi			
							17.6% Relaxation (Meditation)			
							7% Acupuncture			
12	Ali et al. (Ali et al., 2015)	2015	Cross-sectional study	Malaysia	104	67	40.4% Acupuncture		√	√
							40.4% Massage			
							11.5% TCM			

TCM, traditional Chinese medicine; CAM, complementary and alternative medicine; NaCl, sodium chloride.

**TABLE 3:** Prevalence of adherence and methods of assessment.

No	Authors	Year	Type of Study	Country	Sample Size (n)	Prevalence of Non-adherence (%)	Method of Assessment
1	Chambers et al. (Chambers et al., 2011)	2011	Survey	United Kingdom	180	22.2	Medication Adherence Report Scale (MARS)
2	Sjölander et al. (Sjölander et al., 2013)	2013	Cross-sectional study	Sweden	578	12.5	- Medication Adherence Report Scale (MARS)
3	Cheiloudaki et al. (Cheiloudaki and Alexopoulos, 2019)	2019	Survey	United Kingdom	140	31.4	- Medication Adherence Report Scale (MARS)
4	Han et al. (Han et al., 2020)	2019	Cross-sectional study	Brunei	76	10.5	- Modified based on Morisky scale of Adherence
							- Adherence scale Culig
5	Rohde et al. (Rohde et al., 2019)	2019	Observational cohort study	Ireland	108	30.2	- Medication Adherence Rating Scale (MARS-5)
6	Saade et al. (Saade et al., 2021)	2019	Cross-sectional study	Lebanon	100	17	- Lebanese medication adherence scale (LMAS-14)
7	De Schryver et al. (De Schryver et al., 2005)	2005	Cohort study	Netherlands	3,796	26	- Pill-count method
							- Interview by the neurologist
8	Arif et al. (Arif et al., 2007)	2007	Cross-sectional survey	Pakistan	298	32	- Retrospective medical record chart review
9	Khan et al. (Khan et al., 2010)	2010	Cohort study	Canada	3,571	38	- Prescription claims data (home inventory, pill count and serum measures of drug)
10	Bushnell et al. (Bushnell et al., 2011)	2011	Cohort study	US	2,457	36	Adherence Evaluation After Ischemic stroke–Longitudinal (AVAIL) Registry
11	Kronish et al. (Kronish et al., 2013)	2012	Cross-sectional study	US	600	40	8-item Morisky Medication Adherence Questionnaire
12	Ostergaard et al. (Østergaard et al., 2012)	2012	Cohort study	Denmark	503	36	Medication Possession Ratio (MPR)
13	Wei et al. (Wei et al., 2017)	2017	Cross-sectional study	China	313	51	Medicine Adherence Report Scale (MARS)
14	Kim et al. (Kim et al., 2018)	2017	Retrospective cohort study	Korea	8,001	15	Proportion of days covered (PDC) for a period of 1 year
	Statin only in stroke patients						
15	Jiang et al. (Jiang et al., 2017)	2013	Cohort Study	China	18,344	53.8	Pharmacy refills

**TABLE 4:** Factors associated with medication non-adherence.

No	Authors	Year	Type of Study	Country	Patient-Related Factors (Socio-Economic, Perceptions and Belief)	Illness-Related Factors (Severity of Illness and Frequency of Symptoms)	Medication-Related Factors (Number of Daily Doses, Efficacy, and Side Effects), Cost	Physician-Related Factors (Patient-Physician Relationship)
1	Chambers et al. (Chambers et al., 2011)	2011	Cross-sectional survey	United Kingdom	Lack of knowledge Forgetfulness	—	Side effect of medication	Lack of support from health professionals
2	Sjölander et al. (Sjölander et al., 2013)	2013	Cross-sectional survey	Sweden	Lack of confidence in medication 9.7%	—	—	—
3	Cheiloudaki et al. (Cheiloudaki and Alexopoulos, 2019)	2019	Cross-sectional survey	United Kingdom	Patient’s perception of medication necessity	—	—	Doctor–patient communication
					Patient living alone 56.3%			
4	Han et al. (Han et al., 2020)	2019	Cross-sectional study	Brunei	Lack of knowledge	—	Complex medication regimen 9.1%	- Doctors change the prescription 1.3%
5	Rohde et al. (Rohde et al., 2019)	2019	Observational cohort study	Ireland	Lack of knowledge	—	—	—
6	Saade et al. (Saade et al., 2021)	2019	Cross-sectional study	Lebanon	—	—	Side effects	—
							Cost	
7	De Schryver et al. (De Schryver et al., 2005)	2005	Cohort study	Netherlands	—	Less severity of the symptoms	Side effects	—
8	Arif et al. (Arif et al., 2007)	2007	Cross-sectional survey	Pakistan	—	No improvement in condition	—	Doctors change the prescription
9	Khan et al. (Khan et al., 2010)	2010	Cohort study	Canada	—	—	Complex medication regimen	—
10	Bushnell et al. (Bushnell et al., 2011)	2011	Cohort study	US	—	—	—	Doctors change the prescription
11	Kronish et al. (Kronish et al., 2013)	2012	Cross-sectional study	US	—	—	Side effects	Lack of trust in healthcare provider
								Difficulty accessing healthcare
12	Ostergaard et al. (Østergaard et al., 2012)	2012	Cohort study	Denmark	—	—	—	—
13	Wei et al. (Wei et al., 2017)	2017	Cross-sectional study	China	Concerns about their medication	—	Side effects	—
14	Kim et al. (Kim et al., 2018)	2017	Retrospective cohort study	Korea	—	—	Side effects	—
15	Jiang et al. (Jiang et al., 2017)	2013	Cohort study	China	Forgetfulness	—	—	—

**TABLE 5:** CAM usage and adherence among stroke patients.

No	Authors	Sample Size	Country	Year	Prevalence of Adherence n (%)	% Of CAM Usage n (%)	Types of CAM Used	Mind and Body Practice	Natural Products	Alternative/Traditional Medicine
1	Johnson et al. (Johnson et al., 2012)	N = 48	New Zealand	2010	44–48 (91–100%) adherence	11 (21–23%) of natural/herbal remedy	Natural or herbal remedy	√	√	√
2	Kim et al. (Kim et al., 2020)	N = 250	Korea	2006	183 (73%) adherence	0 (0%)	—	—	—	—
3	Karuniawti et al. (Karuniawati et al., 2017)	N = 165	Indonesia	2017	74 (45%) adherence	5 (3.3%)	—	—	—	—
4	Alhawsawi et al	N = 152	Saudi Arabia	2020	90 (59.2%)	29.61% Cauterization	Cauterization Quran recitation	√	—	—
						28.95% Quran recitation				

### Prevalence and Types of Complementary Alternative Medicine Usage

Based on the 12 studies identified, the prevalence of CAM use was between 12 and 67% (Table 2), (Lee et al., 2004; Shah et al., 2008; Shin et al., 2008; Chang et al., 2011; Liao et al., 2012; Pandian et al., 2012; Ali et al., 2015; Kadir et al., 2015; Chang et al., 2016; Teo et al., 2016; Weng et al., 2016; Yeh et al., 2017) The use of CAM in developing countries was higher than that in developed countries. The most prevalent types of CAM reported in these studies were traditional Chinese medicine (TCM), acupuncture, herbal medicine, and Ayurveda.

In developed countries such as the United States, Singapore, Korea, and Taiwan, the prevalence of CAM usage among stroke patients ranged between 12 and 62.3% (Chang et al., 2016; Chang et al., 2011; Lee et al., 2004; Liao et al., 2012; Shah et al., 2008; Shin et al., 2008; Teo et al., 2016; Weng et al., 2016; Yeh et al., 2017). On the other hand, in developing countries, such as India and Malaysia, the prevalence of CAM usage was between 36.3 and 67%, respectively (Ali et al., 2015; Pandian et al., 2012; Kadir et al., 2015). In countries like Singapore, the use of CAM in cardiovascular disease, which includes stroke patients, ranged between 36 and 64% (Table 2), (Teo et al., 2016) This is due to the fact that CAM’s economic worth is growing as a choice of medicine for a range of healthcare demands (Azaizeh et al., 2010).

**FIGURE 2:**
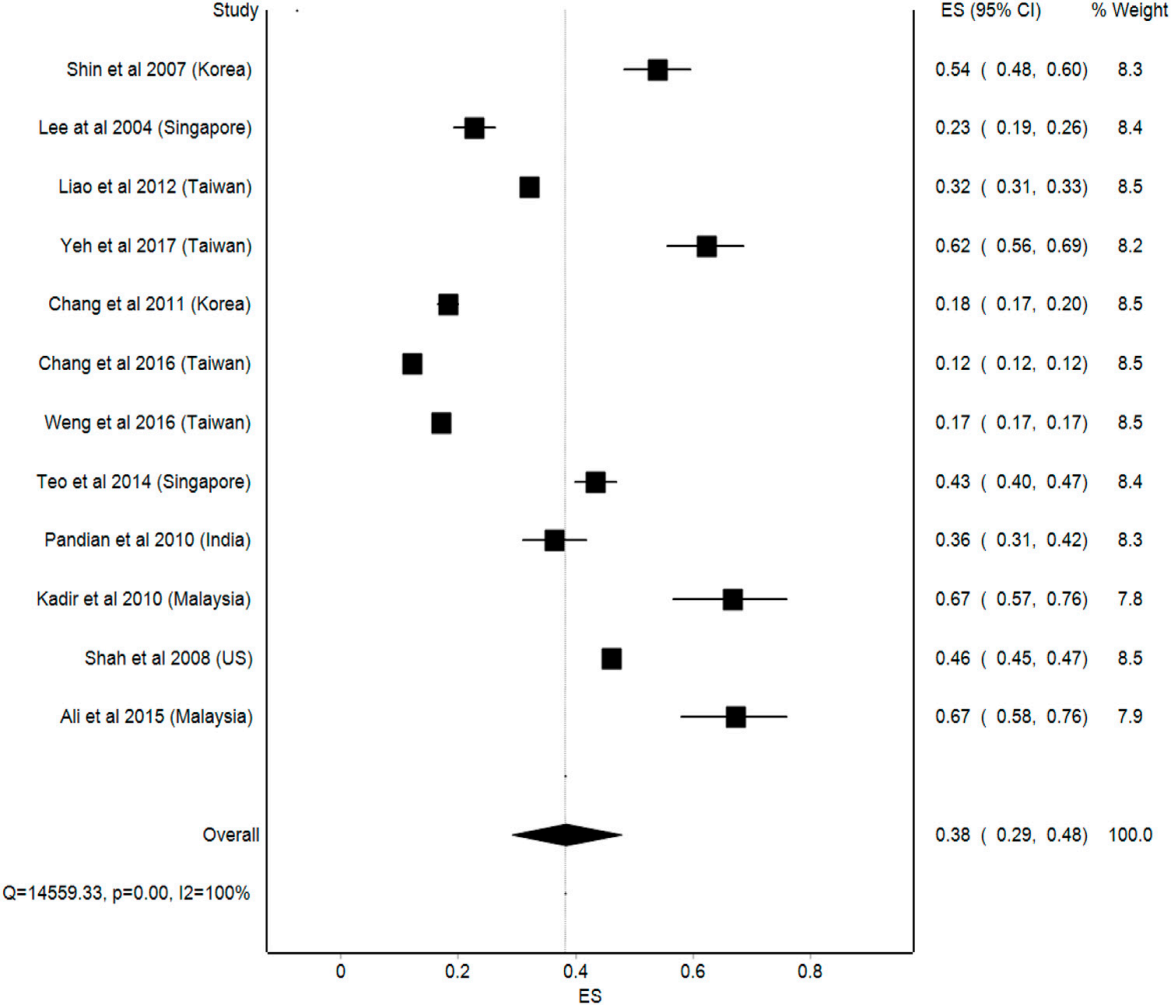
Pooled prevalence of complementary and alternative medicine in stroke patients.

Some types CAM are more regionally unique and may differ between nations. For example, in Korea and Malaysia, herbal and plant-based remedies have become increasingly popular (Ekor, 2014). Studies conducted in Taiwan and Singapore have revealed the use of Traditional Chinese Medicine as the most frequently used type of CAM among stroke patients (Liao et al., 2012). This can be attributed to the practice of Chinese religion in the majority in these countries. One study in India reported the wide use of ayurvedic massage, herbal medicines, and homeopathy (Pandian et al., 2012). Additionally, other non-conventional treatments for stroke have been reported, including witchcraft, opium, marma therapy, and reiki therapy (also known as palm healing), siddha medicine, acupuncture, and unani, which incorporates the four basic elements–earth, air, water, and fire (Pandian et al., 2012). Interestingly, the types of CAMs widely reported in Malaysian studies were the use of vitamins, herbs, and visiting traditional healers (Ali et al., 2015; Kadir et al., 2015). This broad range of CAM can be linked to the multiracial and multicultural diversity of the Malaysian population (Jasamai et al., 2017). Although the pooled prevalence of CAM use among stroke patients was 38% (95%CI: 29%, 48%), a high statistical heterogeneity was observed in this analysis (I^2^ = 100%) (Figure 2).

### Subgroup Analysis

#### Level of Income

**FIGURE 3:**
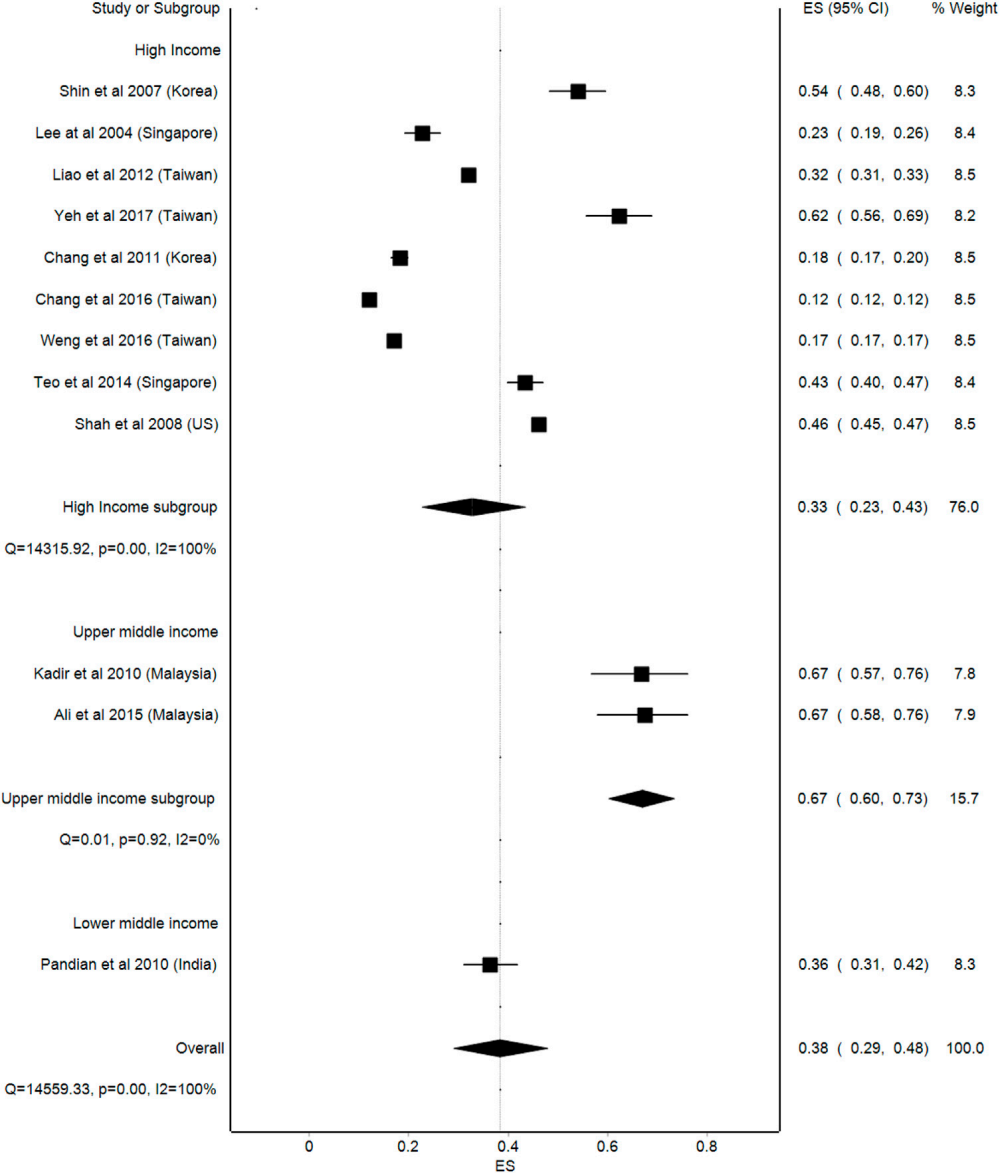
Prevalence proportion of CAM use by level of income.

As for the income level of the countries, a comparatively higher prevalence of CAM usage was observed in upper middle-income countries, where the prevalence was 67% (95%Cl: 60–73, *p* = 0.92) compared to lower middle-income countries, with a prevalence of 38% (95%Cl: 29–48, *p* < 0.001). Meanwhile, the lowest prevalence of CAM usage was among the high-income countries, with a prevalence of 33% (95%Cl: 23–43, *p* < 0.001). Similarly, a high statistical heterogeneity was observed in the analysis (I^2^ = 100%) (Figure 3).

### Factors and Reasons Associated With Complementary Alternative Medicine Use

Among the 12 papers identified, eleven papers reported on the reasons for CAM use. The most commonly reported factors included suggestions from family members and the cultural beliefs of stroke patients (Lee et al., 2004; Shin et al., 2008; Chang et al., 2011; Pandian et al., 2012). These patients believed that CAM usage had more beneficial effects and was easier to obtain than regular treatment (Liao et al., 2012; Yeh et al., 2017). This may be because herbal medication is a natural resource, which is assumed to be safe for use. Stroke patients are also afraid of the side effects of conventional stroke medications, and will therefore look for alternatives, including CAM (Chang et al., 2016; Zou et al., 2018).

### Prevalence of Medication Non-adherence Among Stroke Patients

**FIGURE 4:**
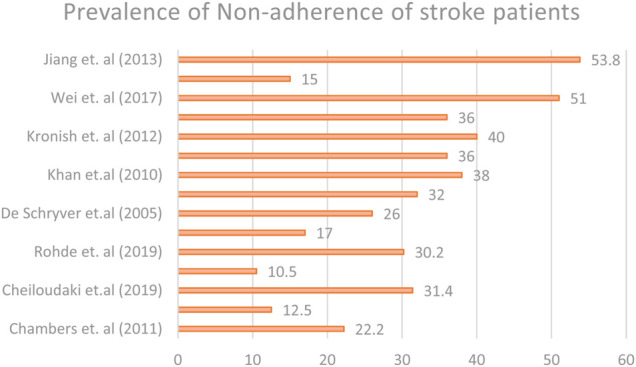
Prevalence of non-adherence.

The prevalence of non-adherence in 15 studies ranged from 10.5 to 53.8%, as shown in Figure 4. Two studies stated that the non-adherence level in the United Kingdom was 22.2 and 31.4%, while two studies from the United States reported a non-adherence level of 36 and 40%. Another two studies from China reported a non-adherence level of 51 and 53.8%. Meanwhile, in countries such as Korea, Lebanon, and Pakistan, the non-adherence levels among stroke patients were 15, 17, and 32%, respectively, which is quite low compared to developed countries, such as the United Kingdom, the United States, and China. In developed countries, such as Denmark, Canada, Sweden, Brunei, Ireland, and the Netherlands, the prevalence of non-adherence among stroke patients was 36, 38, 12.5, 10.5, 30.2, and 26%, respectively.

Four different adherence assessment methods were used across the studies included.(Chambers et al., 2011; Sjölander et al., 2013; Cheiloudaki and Alexopoulos, 2019; Rohde et al., 2019; Han et al., 2020; Saade et al., 2021). The Medication Adherence Report Scale (MARS) was used in most of the studies surveys (Chambers et al., 2011; Sjölander et al., 2013; Cheiloudaki and Alexopoulos, 2019), (Rohde et al., 2019) Meanwhile, other studies used different methods to assess the adherence of stroke patients to their medications. In contrast, Han et al. used the Morisky scale of adherence along with the Adherence scale Culig, while Kronish et al. used the Morisky scale of adherence to assess the adherence of stroke patients (Han et al., 2020).

There were also studies that assessed the prevalence of non-adherence level of stroke patients by collecting medication prescription claim data from the registries of healthcare providers. Moreover, several studies have assessed the adherence of stroke patients by using objective measures, such as the medication possession ratio (MPR) and the pill count method. The prevalence of non-adherence and methods of assessment across all six studies is listed in Table 3.

**FIGURE 5:**
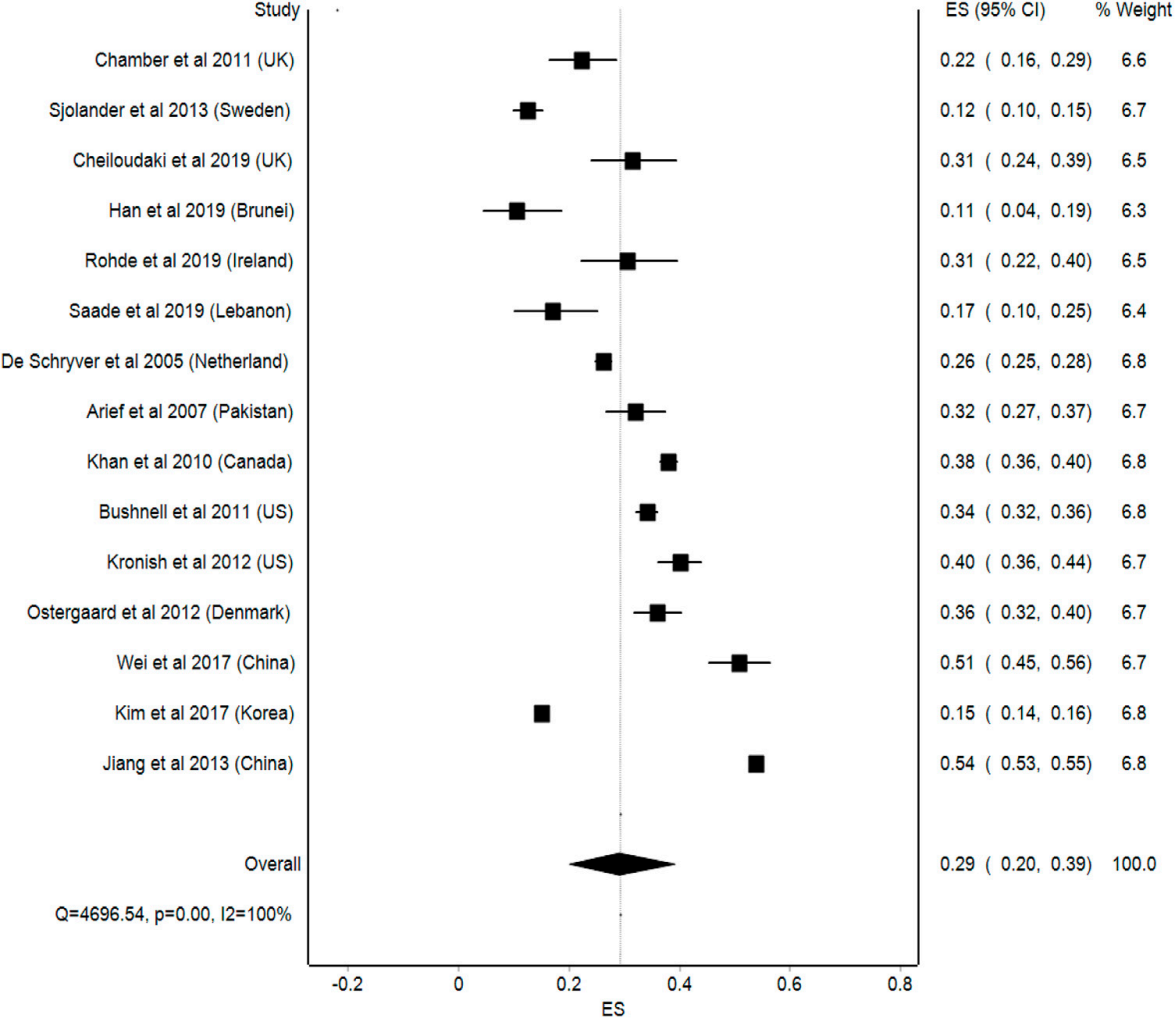
Pooled prevalence of medication adherence in stroke patients.

The pooled prevalence of medication adherence among stroke patients was 29% (95%CI: 20–39%). However, the statistical heterogeneity in this analysis was high, with an I^2^ value of 100% (Figure 5).

### Subgroup Analysis

#### Level of Income

**FIGURE 6:**
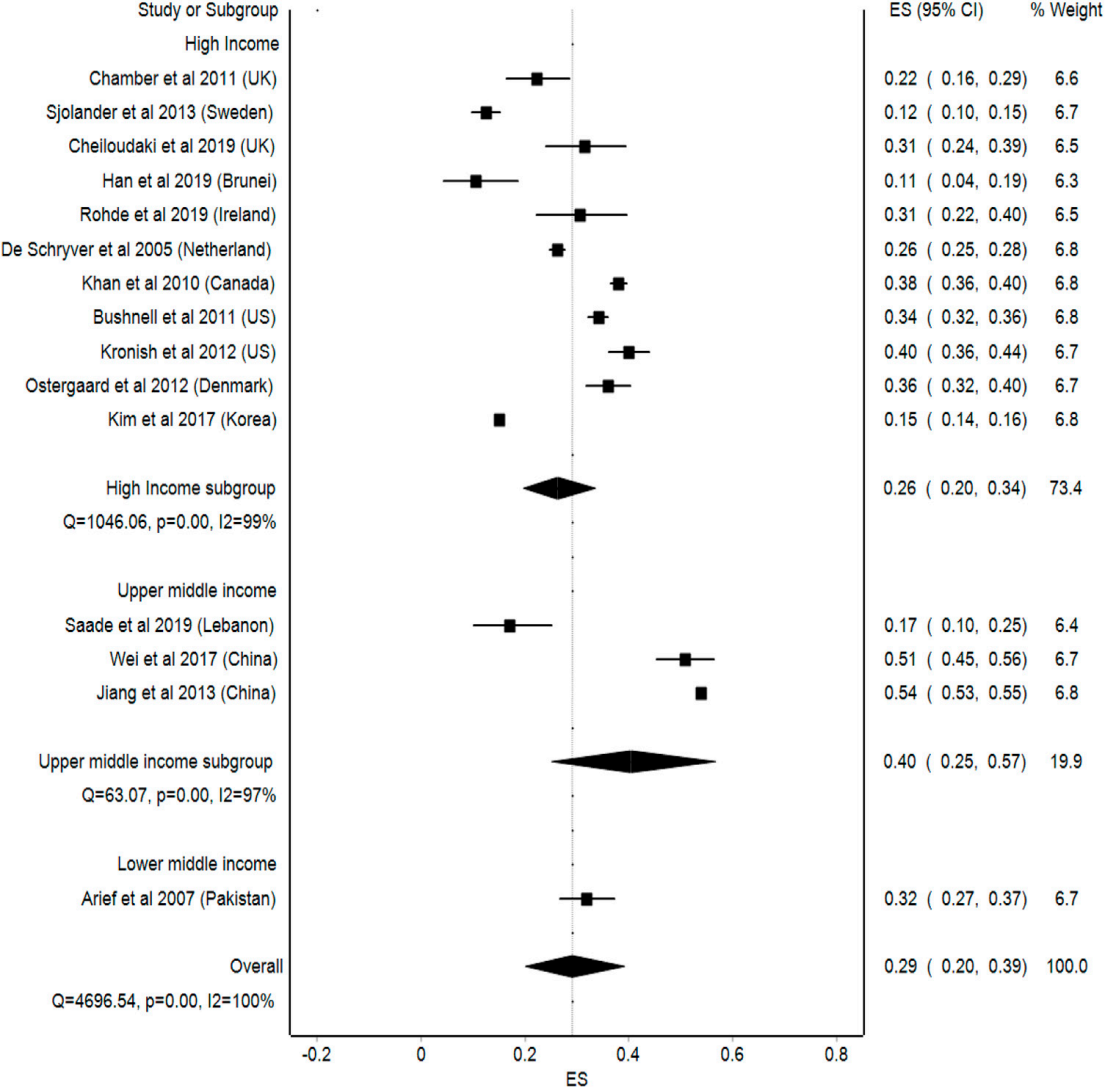
Meta-analysis of medication non-adherence by level of income.

Upper middle-income countries had a higher prevalence of medication non-adherence, which was 40% (95%Cl: 25–57, *p* < 0.001) compared to lower middle-income countries 29% (95%Cl: 20–39, *p* < 0.001) and high-income countries with an average of 26% of non-adherence among stroke patients (95%Cl: 20–34, *p* < 0.001). The statistical heterogeneity observed in the analysis was high (I^2^ = 100%) (Figure 6).

#### Regions

**FIGURE 7:**
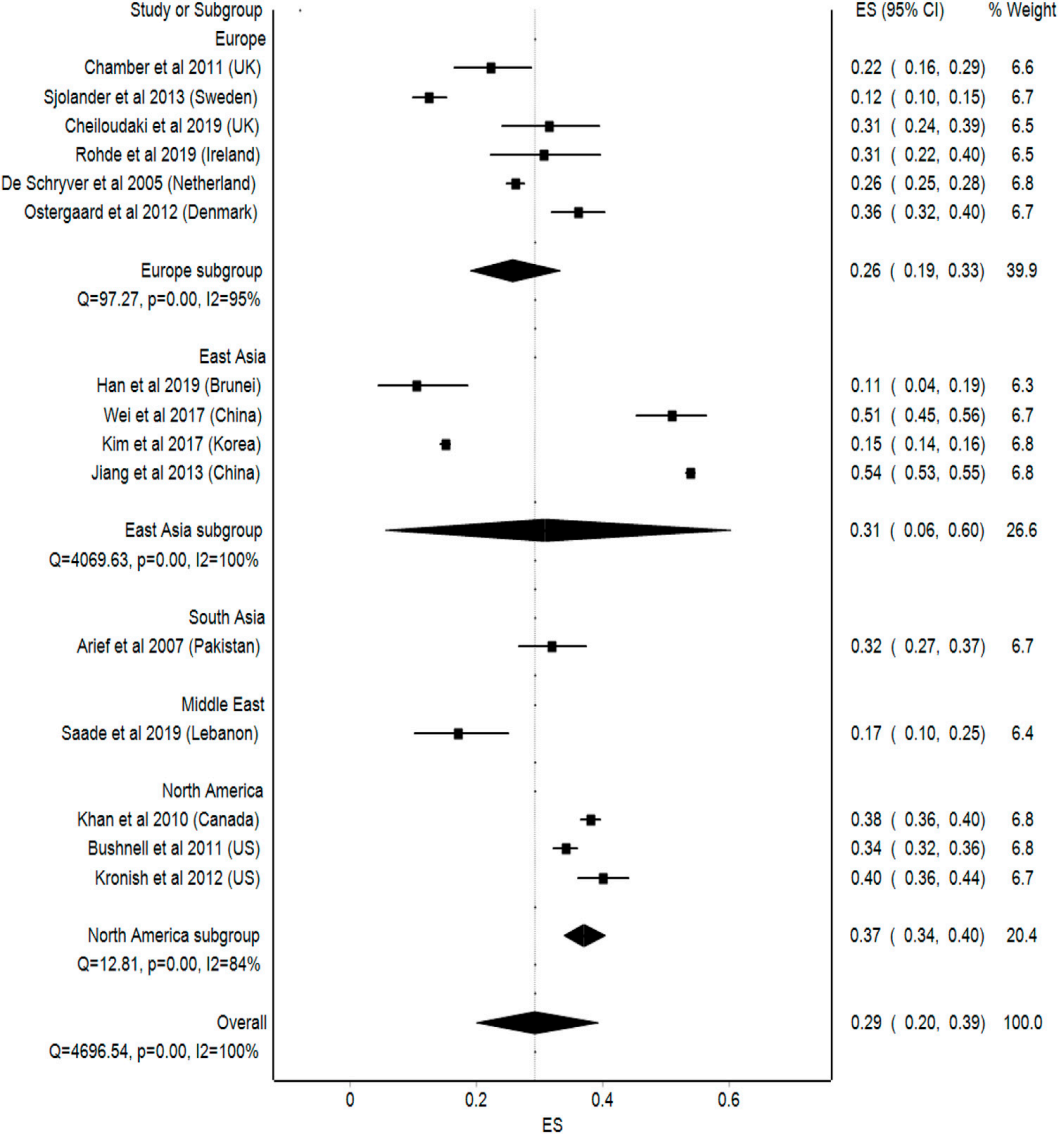
Meta-analysis of medication non-adherence by regions.

As for the regional subgroup analysis, North American countries such as Canada and the United States, had the highest average of non-adherence among stroke patients, which is 37% (95%Cl: 34–40, *p* < 0.001), followed by South Asian countries with an average of 32% (95%Cl: 27–37, *p* < 0.001), East Asian countries with an average of 31% (95%Cl: 6–60, *p* < 0.001), and European countries with an average of 26% (95%Cl: 19–33, *p* < 0.001). Meanwhile, Middle Eastern countries, such as Lebanon, had the lowest average non-adherence among stroke patients, which was 17% (95%Cl: 10–25, *p* < 0.001). This analysis showed a high statistical heterogeneity, where I^2^ = 100% (Figure 7).

### Factors Associated With Non-adherence

The factors associated with medication non-adherence across the 15 studies are outlined in Table 4. In seven studies, patient perception and beliefs, knowledge gap, and forgetfulness when taking medication were the main causes of medication non-adherence among stroke patients (Chambers et al., 2011; Sjölander et al., 2013; Cheiloudaki and Alexopoulos, 2019; Rohde et al., 2019; Han et al., 2020). Other factors, such as medication side effects, cost of the medication, complex medication regimens, and lack of support from health professionals, were also reported to cause stroke medication non-adherence. (Chambers et al., 2011; Sjölander et al., 2013; Cheiloudaki and Alexopoulos, 2019; Rohde et al., 2019; Han et al., 2020; Saade et al., 2021).

#### Patient-Related Factors

In seven of the included studies, the most common factors associated with patient-related factors were the lack of understanding of patients regarding their medication, patient’s confidence regarding their medication, and forgetfulness (Chambers et al., 2011; Sjölander et al., 2013; Cheiloudaki and Alexopoulos, 2019; Rohde et al., 2019; Han et al., 2020). Han et al. reported that non-adherent patients believed their current treatment to be less useful (*p* = 0.001), (Han et al., 2020). Depression was associated with poor adherence in one of the studies, which reported that depression or sadness caused patients to be non-adherent (*p* = 0.001), (Han et al., 2020). Two studies reported forgetfulness as a reason for stroke medication non-adherence, (Chambers et al., 2011; Jiang et al., 2017). The latter is mainly due to the lack of a fixed routine among stroke patients, which causes them to forget to take the medicine on time.

#### Illness-Related Factors

Among the 15 studies that assessed the adherence of stroke patients towards their medication, only one study found that illness-related factors, such as no improvement in patient’s condition, were the reasons for stroke patients’ non-adherence. In this case, patients discontinued their medication because they did not observe any improvement in their condition.

#### Medication-Related Factors

Eight of the studies stated medication-related factors as one of the reasons for non-adherence among stroke patients (De Schryver et al., 2005; Chambers et al., 2011; Kronish et al., 2013; Kim et al., 2017; Wei et al., 2017; Han et al., 2020; Khan et al., 2020; Saade et al., 2021). The most significant medication-related factors associated with non-adherence included complex medication regimens, medication side effects, polypharmacy, and high costs (Khan et al., 2010; Chambers et al., 2011; Kronish et al., 2013; Kim et al., 2017; Wei et al., 2017; Han et al., 2020; Saade et al., 2021). Han et al. reported that patients who received monotherapy showed high treatment adherence, while patients assigned to a complex medication regimen showed moderate-to-low adherence (Han et al., 2020).

#### Physician-Related Factors

Six of the 15 studies reported physician-related factors as one of the reasons for non-adherence. Cheiloudaki et al. reported the impact of the patient-physician relationship and found that trust in doctors was less prevalent among non-adherent patients compared to that in adherent patients (Cheiloudaki and Alexopoulos, 2019). Furthermore, non-adherent participants attributed lack of support from healthcare professionals and frequent changes in their prescription as reason of non-adherence (Arif et al., 2007; Bushnell et al., 2011; Chambers et al., 2011; Han et al., 2020). Moreover, a lack of trust in healthcare providers and difficulty accessing healthcare facilities were also contributing factors to non-adherence among stroke patients (Kronish et al., 2013).

### Effect of Complementary Alternative Medicine Usage on Adherence to Medication Among Stroke Patients

Although clinical evidence regarding the association between stroke medication non-adherence and CAM use is scarce, four studies have assessed the adherence of stroke patients to CAM use. Johnson et al. assessed adherence to stroke medication and CAM use and found that 7% of non-adherent patients were using CAM as a treatment for stroke (Johnson et al., 2012). Similarly, Karuniawati et al. reported that 29% were non-adherent to their medication regimen and 3.3% of them preferred to use CAM (Karuniawati et al., 2017). This study concluded that patients tended to stop taking their medication after feeling better. In contrast, Kim et al. reported that there was no association between CAM use and non-adherence to stroke medication (Kim et al., 2018). Moreover, a study done by Alhawsawi et al. have reported that 59.2% of the stroke patients were non-adherent towards their medications and 29.61% of the stroke patients had used CAM.

### Quality of Studies Included in the Analysis

The quality of the studies included in the analysis was variable. For example, the response rate in the studies ranged between 14.4 and 100%. Studies by Zou et al. and Han et al. did not report response rates. (Zou et al., 2018; Han et al., 2020). The manner in which participants were recruited and the sample size were not clearly reported in two studies (Chambers et al., 2011; Han et al., 2020). The types of CAM used were not explained in one study (Zou et al., 2018). The adherence of patients to medication was mostly assessed using the Medication Adherence Report Scale (MARS). (Chambers et al., 2011; Sjölander et al., 2013; Cheiloudaki and Alexopoulos, 2019; Rohde et al., 2019). Moreover, Ostergaard et al., who studied the prevalence of non-adherence, did not collect data on the reasons for non-adherence among stroke patients. All studies clearly explained how medication adherence was measured (Han et al., 2020; Saade et al., 2021).

## Discussion

In this study, the usage trends and effects of CAM on adherence to stroke treatment in stroke patients was reviewed. A total of 31 studies were included, within which the poor response rate in the surveys was a specific concern. Further issues with CAM surveys have been attributed to the fact that there is no widely agreed classification of CAM. Multiple approaches have been used to measure adherence in different studies. For many factors, self-reported questionnaires may overestimate adherence: first, they can depend on patients’ own understanding or recollection of what guidance has been provided; second, patients may appear to record higher levels of adherence in order to please healthcare professionals or prevent personal embarrassment (Tiv et al., 2012). Over time, the popularity of CAM treatments for stroke in developed nations has grown, at times in accordance with changes in the healthcare environment, the quality of schooling, and evolving societal values.

The most prevalent types of CAM usage by stroke patients reported in the studies reviewed were the use of herbal products, traditional Chinese medicine, massage, vitamins, and acupuncture. Meanwhile, the use of CAM has been studied among different sample populations including breast cancer, asthma, bone cancer, chronic diseases, and epilepsy, and the prevalence of CAM use was found to be 70.7, 61.1, 68, 63.9, and 26.7% respectively.^26-30^ A wide variation in the prevalence of CAM use was observed in this review, between 12 and 67%. The meta-analysis, which was carried out on the prevalence of the CAM use data, showed high statistical variation in CAM usage among stroke patients across different studies. The subgroup analysis across countries’ income levels showed that upper middle-income countries, such as Malaysia, had the highest prevalence of CAM usage. This may be due to the multicultural and multiracial diversity of Malaysian citizens, which allows them to try different types of CAM that are available in Malaysia. (Jasamai et al., 2017).

Moreover, the prevalence of stroke medication non-adherence was reported in 6 of the studies, ranging between 5.7 and 30.2%. Similarly, meta-analysis showed a high statistical heterogeneity among the studies. Subgroup analysis across income levels found that high-income countries had the lowest average of non-adherence among stroke patients. This may be due to a higher level of disease awareness among stroke patients and better healthcare systems across high-income countries compared to upper-middle-income countries and lower-middle-income countries. Furthermore, the assessment methods used in these studies were mainly subjective (patient-reported questionnaires) and tended to overestimate patient adherence. Patient compliance can be estimated more reliably using a mixed-method methodology (i.e., using subjective and quantitative evaluation tools).

Forgetfulness, specific beliefs about medications, lack of patient knowledge on medication, fear of side effects, complex medication regimens, and a poor doctor-patient relationship were the most frequently reported factors associated with non-adherence to stroke medication. Similar findings were reported in a study in which patients on poly therapy were more likely to forget to take their medication (Han et al., 2020).

Next, the most frequently reported reasons for non-adherence among stroke patients were lack of knowledge, complex medication regimens, and poor relationships between physicians and patients. These factors are likely to contribute to a shift toward the use of CAM among patients with stroke (Johnson et al., 2012; Kim et al., 2018). Studies have shown that many stroke patients tend to concurrently use both CAM and stroke medication. This may be attributed to appealing marketing tactics employed by herbal and vitamin firms, who make promises about the advantages of complementary and alternative medicine (CAM), attracting these patients- However, there is a lack of clinical evidence regarding their efficacy in preventing the worsening of stroke or their safety profiles.

Pharmacists could conduct counselling and educational programs to help patients improve medication adherence and change their false perceptions about the etiology of stroke. According to one report, pharmacists should play a more active role in ensuring proper clinical knowledge is disseminated to patients (Islahudin and Tan, 2013). These findings are consistent with those of a study in which the compliance rate was high among patients with good knowledge about their disease (Omar and San, 2014). Moreover, the use of various compliance aids, such as pill boxes, medication reminders, and combination therapy (where possible) to reduce the number of pills, can improve adherence (Vervloet et al., 2012). Subsequently, in order to enhance patients’ understanding of their medication, pharmacists could counsel patients, providing details of drug names, doses, routes of administration, frequency of dosing, and duration of treatment (Osterberg and Blaschke, 2005). This information is important because it would promote the correct self-administration of medication among patients. As a result, counselling patients could mitigate adverse effects associated with medication non-adherence, thereby ensuring successful therapy.

## Limitations

With regards to the limitations of this work, there seems to be limited evidence on stroke medication non-adherence as a result of CAM usage. Although many studies have reported the reasons for CAM usage among stroke patients, among the 20 studies included in this review, the association between stroke medication adherence and CAM use was only assessed in three studies (Johnson et al., 2012; Karuniawati et al., 2017; J.; Kim et al., 2018).

Further studies investigating the resultant effects of CAM use by stroke patients will be needed to determine whether the treatment under consideration has a positive or negative impact on patients’ health conditions and adherence to medication.

## Conclusion

The prevalence of CAM usage among stroke patients has been reported to range between 12 and 62.3%. Meanwhile, the prevalence of medication non-adherence among stroke ranged from 10.5 to 31.4%. Medication non-adherence is thought to result from a limited understanding of clinical treatments, fear of side effects, poor patient-physician relationships, complex medication regimens, and the use of CAMs instead of stroke medication. Due to the lack of published studies, evaluating the impact of CAM usage on adherence to medication among stroke patients is warranted to gain further.

## Data Availability

The original contributions presented in the study are included in the article/supplementary materials, further inquiries can be directed to the corresponding authors.

## References

[B1] Alhawsawi T. Alghamdi M. Albaradei O. Zaher H. Balubaid W. Alotibi H. A. (2020). Complementary and Alternative Medicine Use Among Ischemic Stroke Survivors in Jeddah, Saudi Arabia. Nsj 25 (5), 362–368. 10.17712/nsj.2020.5.20200088 PMC8015602 33459284

[B2] Ali M. F. Aziz A. F. A. Rashid M. R. Man Z. C. Amir A. A. Lim Y. S. (2015). Usage of Traditional and Complementary Medicine (T & CM): Prevalence, Practice and Perception Among Post Stroke Patients Attending Conventional Stroke Rehabilitation in A Teaching Hospital in Malaysia. Med. J. Malays. 70 (1), 18–23. 26032524

[B3] Arif H. Aijaz B. Islam M. Aftab U. Kumar S. Shafqat S. (2007). Drug Compliance after Stroke and Myocardial Infarction: A Comparative Study. Neurol. India 55 (2), 130–135. 10.4103/0028-3886.32783 17558116

[B4] Aydin S. Bozkaya A. O. Mazicioğlu M. M. Gemalmaz A. Özçakir A. Öztürk A. (2008). What Influences Herbal Medicine Use?-Prevalence and Related Factors. Turkish J. Med. Sci. 38 (5), 455–463.

[B5] Azaizeh H. Saad B. Cooper E. Said O. (2010). Traditional Arabic and Islamic Medicine, a Re-emerging Health Aid. Evid. Based Complement. Altern. Med. 7 (4), 419–424. 10.1093/ecam/nen039 PMC2892355 18955344

[B6] Barendregt J. J. Doi S. A. Lee Y. Y. Norman R. E. Vos T. (2013). Meta-analysis of Prevalence. J. Epidemiol. Community Health 67 (11), 974–978. 10.1136/jech-2013-203104 23963506

[B7] Barnes P. M. Bloom B. Nahin R. L. (2008). Complementary and Alternative Medicine Use Among Adults and Children; United States, 2007. Natl. Health Stat. Rep. 12, 1–23. 19361005

[B8] Bushnell C. D. Olson D. M. Zhao X. Pan W. Zimmer L. O. Goldstein L. B. (2011). Secondary Preventive Medication Persistence and Adherence 1 Year after Stroke. Neurology 77 (12), 1182–1190. 10.1212/WNL.0b013e31822f0423 21900638 PMC3265047

[B9] Chambers J. A. O'Carroll R. E. Hamilton B. Whittaker J. Johnston M. Sudlow C. (2011). Adherence to Medication in Stroke Survivors: A Qualitative Comparison of Low and High Adherers. Br. J. Health Psychol. 16 (3), 592–609. 10.1348/2044-8287.002000 21199537

[B10] Chang H. Kwon Y. D. Yoon S. S. (2011). Use of Acupuncture Therapy as a Supplement to Conventional Medical Treatments for Acute Ischaemic Stroke Patients in an Academic Medical Centre in Korea. Complement. Ther. Med. 19 (5), 256–263. 10.1016/j.ctim.2011.07.003 21944655

[B11] Chang C. C. Lee Y. C. Lin C. C. Chang C. H. Chiu C. D. Chou L. W. (2016). Characteristics of Traditional Chinese Medicine Usage in Patients with Stroke in Taiwan: A Nationwide Population-Based Study. J. Ethnopharmacol. 186, 311–321. 10.1016/j.jep.2016.04.018 27090345

[B12] Cheiloudaki E. Alexopoulos E. C. (2019). Adherence to Treatment in Stroke Patients. Int. J. Environ. Res. Public Health 16 (2), 196. 10.3390/ijerph16020196 PMC6351941 30641978

[B13] De Schryver E. L. van Gijn J. Kappelle L. J. Koudstaal P. J. Algra A. (2005). Non-adherence to Aspirin or Oral Anticoagulants in Secondary Prevention after Ischaemic Stroke. J. Neurol. 252 (11), 1316–1321. 10.1007/s00415-005-0858-0 15868068

[B14] Ekor M. (2014). The Growing Use of Herbal Medicines: Issues Relating to Adverse Reactions and Challenges in Monitoring Safety. Front. Pharmacol. 4, 177. 10.3389/fphar.2013.00177 24454289 PMC3887317

[B15] Fox P. Coughlan B. Butler M. Kelleher C. (2010). Complementary Alternative Medicine (CAM) Use in Ireland: A Secondary Analysis of SLAN Data. Complement. Ther. Med. 18 (2), 95–103. 10.1016/j.ctim.2010.02.001 20430292

[B16] Greenhouse W. J. Meyer B. Johnson S. L. (2000). Coping and Medication Adherence in Bipolar Disorder. J. Affect Disord. 59 (3), 237–241. 10.1016/s0165-0327(99)00152-4 10854641

[B17] Han Y. K. Rajabalaya R. Yassin D. H. N. B. P. H. M. David S. R. (2020). Medication Adherence in Stroke Patients in Brunei Darussalam Public Hospital: The Cross‐sectional Study Associated with Chronic Diseases, Life Style and Potential Barriers. J. Pharm. Health Serv. Res. 11 (2), 127–132. 10.1111/jphs.12335

[B18] Higgins J. P. Thompson S. G. (2002). Quantifying Heterogeneity in a Meta-Analysis. Stat. Med. 21 (11), 1539–1558. 10.1002/sim.1186 12111919

[B19] Islahudin F. Tan S. (2013). Medication Knowledge and Adherence in Nephrology Patients. Int. J. Pharm. Bio Sci. 3 (1), 459–466.

[B20] Jasamai M. Islahudin F. Samsuddin N. F. (2017). Attitudes towards Complementary Alternative Medicine Among Malaysian Adults. J. Appl. Pharm. Sci. 7 (06), 190–193. 10.7324/JAPS.2017.70627

[B21] Jeong M. J. Lee H. Y. Lim J. H. Yun Y. J. (2016). Current Utilization and Influencing Factors of Complementary and Alternative Medicine Among Children with Neuropsychiatric Disease: A Cross-Sectional Survey in Korea. BMC Complement. Altern. Med. 16 (1), 91–98. 10.1186/s12906-016-1066-4 26931188 PMC4774171

[B22] Jiang Y. Yang X. Li Z. Pan Y. Wang Y. Wang Y. (2017). Persistence of Secondary Prevention Medication and Related Factors for Acute Ischemic Stroke and Transient Ischemic Attack in China. Neurol. Res. 39 (6), 492–497. 10.1080/01616412.2017.1312792 28420316

[B23] Johnson C. Lane H. Barber P. A. Charleston A. (2012). Medication Compliance in Ischaemic Stroke Patients. Intern Med. J. 42 (4), e47–52. 10.1111/j.1445-5994.2010.02209.x 20214689

[B24] Kadir A. A. Hamid A. H. Mohammad M. (2015). Pattern of Complementary and Alternative Medicine Use Among Malaysian Stroke Survivors: A Hospital-Based Prospective Study. J. Tradit. Complement. Med. 5 (3), 157–160. 10.1016/j.jtcme.2014.11.010 26151028 PMC4488570

[B25] karuniawati h. ikawati z. gofir A. (2017). Adherence to Secondary Stroke Prevention Therapies in Ischemic Stroke Patients at Teaching Hospital in Central Java Indonesia. Congest. heart Fail. 36, 22. 10.22159/ajpcr.2017.v10s2.19478

[B26] Khaku A. S. Tadi P. (2020). Cerebrovascular Disease (Stroke). Treasure Island (FL): StatPearls. [Internet].

[B27] Khan N. A. Yun L. Humphries K. Kapral M. (2010). Antihypertensive Drug Use and Adherence after Stroke: Are There Sex Differences? Stroke 41 (7), 1445–1449. 10.1161/STROKEAHA.110.579375 20508191

[B28] Khan S. U. Singh M. Valavoor S. Khan M. U. Lone A. N. Khan M. Z. (2020). Dual Antiplatelet Therapy after Percutaneous Coronary Intervention and Drug-Eluting Stents: A Systematic Review and Network Meta-Analysis. Circulation 142 (15), 1425–1436. 10.1161/CIRCULATIONAHA.120.046308 32795096 PMC7547897

[B29] Kim J. Lee H. S. Nam C. M. Heo J. H. (2017). Effects of Statin Intensity and Adherence on the Long-Term Prognosis after Acute Ischemic Stroke. Stroke 48 (10), 2723–2730. 10.1161/STROKEAHA.117.018140 28916672

[B30] Kim J. Bushnell C. D. Lee H. S. Han S. W. (2018). Effect of Adherence to Antihypertensive Medication on the Long-Term Outcome after Hemorrhagic Stroke in Korea. Hypertension 72 (2), 391–398. 10.1161/HYPERTENSIONAHA.118.11139 29915019

[B31] Kim G. G. Chae D. H. Park M. S. Yoo S. H. (2020). Factors Influencing 1-Year Medication Adherence of Korean Ischemic Stroke Survivors. Int. J. Behav. Med. 27 (2), 225–234. 10.1007/s12529-020-09854-z 32026290

[B32] Kleindienst N. Greil W. (2004). Are Illness Concepts a Powerful Predictor of Adherence to Prophylactic Treatment in Bipolar Disorder? J. Clin. Psychiatry 65 (7), 966–974. 10.4088/jcp.v65n0713 15291686

[B33] Kronish I. M. Diefenbach M. A. Edmondson D. E. Phillips L. A. Fei K. Horowitz C. R. (2013). Key Barriers to Medication Adherence in Survivors of Strokes and Transient Ischemic Attacks. J. Gen. Intern Med. 28 (5), 675–682. 10.1007/s11606-012-2308-x 23288379 PMC3631079

[B34] Lee G. B. Charn T. C. Chew Z. H. Ng T. P. (2004). Complementary and Alternative Medicine Use in Patients with Chronic Diseases in Primary Care Is Associated with Perceived Quality of Care and Cultural Beliefs. Fam. Pract. 21 (6), 654–660. 10.1093/fampra/cmh613 15531625

[B35] Liao C.-C. Lin J.-G. Tsai C.-C. Lane H.-L. Su T.-C. Wang H.-H. (2012). An Investigation of the Use of Traditional Chinese Medicine in Stroke Patients in Taiwan. Evidence-Based Complementary Altern. Med. 2012, 387164. 10.1155/2012/387164 PMC3530233 23304199

[B36] Linden B. (2020). National Institute for Health and Care Excellence NG128 Stroke and Transient Ischaemic Attack in over 16s: Diagnosis and Initial Management. Br. J. Cardiac Nurs. 15 (9), 1–5. 10.12968/bjca.2020.0121

[B37] Lindsay M. P. Norrving B. Sacco R. L. Brainin M. Hacke W. Martins S. (2019). World Stroke Organization (WSO): Global Stroke Fact Sheet 2019. London, England: SAGE Publications Sage UK. 10.1177/1747493019881353 31658892

[B38] Long A. F. Godfrey M. Randall T. Brettle A. Grant M. J. (2002). HCPRDU Evaluation Tool for Quantitative Studies. Retrieved from http://usir.salford.ac.uk/12969/ .

[B39] MOH (2012). Clinical Practice Guideline : Management of Ischaemic Stroke Malaysia MOH. Available at: https://www.moh.gov.my/moh/resources/Penerbitan/CPG/CARDIOVASCULAR/CPG_Management_of_Ischaemic_Stroke_3rd_Edition_2020_28.02_.2021_.pdf .

[B40] MOH (2019). Discover! Malaysia’s Stroke Care Revolution. Available at: https://www.crc.gov.my/wp-content/uploads/documents/Journal/Discover_Malaysia_Stroke_Care_RevolutionSE.pdf .

[B41] Omar M. S. San K. L. (2014). Diabetes Knowledge and Medication Adherence Among Geriatric Patient with Type 2 Diabetes Mellitus. Malay 36, 53–54.

[B42] O'Neill D. Horgan F. Hickey A. McGee H. (2008). Long Term Outcome of Stroke: Stroke Is a Chronic Disease with Acute Events. Bmj 336 (7642), 461. 10.1136/bmj.39500.434086.1F PMC2258352 18309967

[B43] Osterberg L. Blaschke T. (2005). Adherence to Medication. N. Engl. J. Med. 353 (5), 487–497. 10.1056/NEJMra050100 16079372

[B44] Østergaard K. Hallas J. Bak S. Christensen Rd. Gaist D. (2012). Long-term Use of Antiplatelet Drugs by Stroke Patients: A Follow-Up Study Based on Prescription Register Data. Eur. J. Clin. Pharmacol. 68 (12), 1631–1637. 10.1007/s00228-012-1293-7 22576729

[B45] Pandian J. D. Toor G. Arora R. Kaur P. Dheeraj K. V. Bhullar R. S. (2012). Complementary and Alternative Medicine Treatments Among Stroke Patients in India. Top. Stroke Rehabil. 19 (5), 384–394. 10.1310/tsr1905-384 22982825

[B46] Rhodes P. J. Small N. Ismail H. Wright J. P. (2008). The Use of Biomedicine, Complementary and Alternative Medicine, and Ethnomedicine for the Treatment of Epilepsy Among People of South Asian Origin in the UK. BMC Complement. Altern. Med. 8 (1), 7–14. 10.1186/1472-6882-8-7 18366698 PMC2329602

[B47] Rohde D. Gaynor E. Large M. Mellon L. Bennett K. Williams D. J. (2019). Cognitive Impairment and Medication Adherence Post-stroke: A Five-Year Follow-Up of the ASPIRE-S Cohort. PloS one 14 (10), e0223997. 10.1371/journal.pone.0223997 31622438 PMC6797135

[B48] Saade S. Kobeissy R. Sandakli S. Malaeb D. Lahoud N. Hallit S. (2021). Medication Adherence for Secondary Stroke Prevention and its Barriers Among Lebanese Survivors: A Cross-Sectional Study. Clin. Epidemiol. Glob. Health 9, 338–346. 10.1016/j.cegh.2020.10.007

[B49] Sabaté E. Sabaté E. (2003). Adherence to Long-Term Therapies: Evidence for Action. Geneva, Switzerland: World Health Organization. Available at: http://whqlibdoc.who.int/publications/2003/9241545992.pdf . 14562485

[B50] Scott J. Pope M. (2002). Nonadherence with Mood Stabilizers: Prevalence and Predictors. J. Clin. Psychiatry 63 (5), 384–390. 10.4088/jcp.v63n0502 12019661

[B51] Shah S. H. Engelhardt R. Ovbiagele B. (2008). Patterns of Complementary and Alternative Medicine Use Among United States Stroke Survivors. J. Neurol. Sci. 271 (1-2), 180–185. 10.1016/j.jns.2008.04.014 18485369

[B52] Shaikh S. H. Malik F. James H. Abdul H. (2009). Trends in the Use of Complementary and Alternative Medicine in Pakistan: A Population-Based Survey. J. Altern. Complement. Med. 15 (5), 545–550. 10.1089/acm.2008.0232 19422284

[B53] Shams M. E. Barakat E. A. (2010). Measuring the Rate of Therapeutic Adherence Among Outpatients with T2DM in Egypt. Saudi Pharm. J. 18 (4), 225–232. 10.1016/j.jsps.2010.07.004 23960731 PMC3730985

[B54] Shin Y. I. Yang C. Y. Joo M. C. Lee S. G. Kim J. H. Lee M. S. (2008). Patterns of Using Complementary and Alternative Medicine by Stroke Patients at Two University Hospitals in Korea. Evid. Based Complement. Altern. Med. 5 (2), 231–235. 10.1093/ecam/nem025 PMC2396475 18604256

[B55] Sjölander M. Eriksson M. Glader E.-L. (2013). The Association between Patients’ Beliefs about Medicines and Adherence to Drug Treatment after Stroke: A Cross-Sectional Questionnaire Survey. BMJ open 3 (9), e003551. 10.1136/bmjopen-2013-003551 PMC3787491 24068768

[B56] Teo T. Y. Yap J. Shen T. Yeo K. K. (2016). Complementary and Alternative Medicine Use Amongst Patients with Cardiovascular Disease in Singapore. BMC Complement. Altern. Med. 16 (1), 446–447. 10.1186/s12906-016-1430-4 27825376 PMC5101719

[B57] Tiv M. Viel J. F. Mauny F. Eschwège E. Weill A. Fournier C. (2012). Medication Adherence in Type 2 Diabetes: The ENTRED Study 2007, a French Population-Based Study. PLoS One 7 (3), e32412. 10.1371/journal.pone.0032412 22403654 PMC3293796

[B58] Velligan D. I. Wang M. Diamond P. Glahn D. C. Castillo D. Bendle S. (2007). Relationships Among Subjective and Objective Measures of Adherence to Oral Antipsychotic Medications. Psychiatr. Serv. 58 (9), 1187–1192. 10.1176/ps.2007.58.9.1187 17766564

[B59] Vermeire E. Hearnshaw H. Van Royen P. Denekens J. (2001). Patient Adherence to Treatment: Three Decades of Research. A Comprehensive Review. J. Clin. Pharm. Ther. 26 (5), 331–342. 10.1046/j.1365-2710.2001.00363.x 11679023

[B60] Vervloet M. Linn A. J. van Weert J. C. De Bakker D. H. Bouvy M. L. Van Dijk L. (2012). The Effectiveness of Interventions Using Electronic Reminders to Improve Adherence to Chronic Medication: A Systematic Review of the Literature. J. Am. Med. Inf. Assoc. 19 (5), 696–704. 10.1136/amiajnl-2011-000748 PMC3422829 22534082

[B61] Virani S. S. Alonso A. Aparicio H. J. Benjamin E. J. Bittencourt M. S. Callaway C. W. (2021). Heart Disease and Stroke Statistics-2021 Update: A Report from the American Heart Association. Circulation 143 (8), e254–e743. 10.1161/CIR.0000000000000950 33501848 PMC13036842

[B62] Vrijens B. De Geest S. Hughes D. A. Przemyslaw K. Demonceau J. Ruppar T. (2012). A New Taxonomy for Describing and Defining Adherence to Medications. Br. J. Clin. Pharmacol. 73 (5), 691–705. 10.1111/j.1365-2125.2012.04167.x 22486599 PMC3403197

[B63] Wei L. Champman S. Li X. Li X. Li S. Chen R. (2017). Beliefs about Medicines and Non-adherence in Patients with Stroke, Diabetes Mellitus and Rheumatoid Arthritis: A Cross-Sectional Study in China. BMJ open 7 (10), e017293. 10.1136/bmjopen-2017-017293 PMC5640055 28982826

[B64] Weng S. W. Chen T. L. Yeh C. C. Liao C. C. Lane H. L. Lin J. G. (2016). An Investigation of the Use of Acupuncture in Stroke Patients in Taiwan: A National Cohort Study. BMC Complement. Altern. Med. 16 (1), 321–328. 10.1186/s12906-016-1272-0 27566677 PMC5002127

[B65] Wieland L. S. Manheimer E. Berman B. M. (2011). Development and Classification of an Operational Definition of Complementary and Alternative Medicine for the Cochrane Collaboration. Altern. Ther. Health Med. 17 (2), 50–59. PMC3196853 21717826

[B66] Yeh M. L. Chiu W. L. Wang Y. J. Lo C. (2017). An Investigation of the Use of Traditional Chinese Medicine and Complementary and Alternative Medicine in Stroke Patients. Holist. Nurs. Pract. 31 (6), 400–407. 10.1097/HNP.0000000000000238 29028779

[B67] Zou L. Yeung A. Li C. Chiou S. Y. Zeng N. Tzeng H. M. (2018). Effects of Mind⁻Body Movements on Balance Function in Stroke Survivors: A Meta-Analysis of Randomized Controlled Trials. Int. J. Environ. Res. Public Health 15 (6), 1292. 10.3390/ijerph15061292 PMC6025433 29925770

